# Modelling the Leachability of Strontium and Barium from Stone Building Materials

**DOI:** 10.3390/ma14123403

**Published:** 2021-06-19

**Authors:** Agnieszka Pękala, Michał Musiał

**Affiliations:** 1 Department of Environmental Engineering and Chemistry, Rzeszów University of Technology, al. Powstanców Warszawy 6, 35-959 Rzeszów, Poland; 2 Department of Building Engineering, Rzeszów University of Technology, Poznańska 2, 35-959 Rzeszów, Poland; mmusial@prz.edu.pl

**Keywords:** trace elements, decision system, fuzzy inference, environment, opoka rock

## Abstract

In order that the impact on the environment and human beings can be assessed, it may prove necessary for geochemical research work to entail determinations of concentrations of trace elements in building materials, and it is also likely that this will be a time-consuming and financially-demanding business. Additionally, once basic research has been carried out to determine the mineral composition and structural and textural features, it will then be important to determine concentrations of elements that affect the surrounding natural environment and the health of human beings. This paper thus describes mineralogical and geochemical analyses performed on the stone material that opoka rocks represent. Mineralogical studies have shown that the studied opoka rocks most often have cryptocrystalline silica dispersed among carbonate components. The texture of the rock is slightly porous. Silica in the form of type opal A and CT (cristobalite–tridymite) is the main mineral component of the opoka rocks. Carbonate minerals represented by calcite were an important component in the opoka rocks. Earlier geochemical studies focused on the concentration of Sr and Ba. However, the determination of the leachability of these elements as a function of time is a novelty in this study. Trace elements leached from the material matrix were made subject to determinations. The MATLAB program was used to assess leachability in the cases of both strontium and barium, by reference to the Mamdani–Assilian fuzzy algorithm. The presented work has thus sought to experiment with the use of statistical methods to monitor the effectiveness of geochemical processes taking place over time.

## 1. Introduction

Environmental assessment systems that are deployed in relation to construction materials take into account their impact on the natural environment. Such an assessment will require detailed analysis of a given material’s internal structure, as well as external factors capable of affecting releases of hazardous substances. It is not only composites based on harmful materials that are tested, but also natural stone materials—given the possible presence of harmful components (possibly even of natural origin) in these too. Comprehensive material analysis includes research on the material matrix, in line with knowledge of the physical, chemical and mechanical properties. This is important not only industrially, but above all also ecologically. The impacts of external factors (precipitation, aggressive groundwater and other aggressive liquids) may release additional amounts of components originally associated with a material’s structure. In many countries, work is underway to establish an environmental assessment system for materials used in construction. In these works, attention is paid to the leaching of chemical elements from recycled building aggregates and stone waste materials used in road construction [1,2].

While detailed laboratory tests of building materials are needed, a balanced statistical analysis of the obtained results is also important. During comprehensive studies of the concentration of elements or chemical compounds present in stone building materials, attempts are made to determine the concentration of elements using statistical methods [3]. System identification entails the use of mathematical tools and algorithms to build dynamic models that describe the behaviors of real-world systems on the basis of measurement data [4].

Therefore, it is necessary to create a system that would offer a skillful combination of laboratory tests on the one hand, and expert knowledge and available databases on the other. This would then allow the selection of optimal solutions. In this context, the most used tools are Leach XS and Orchestra [5,6,7,8,9,10,11,12,13,14,15]. In fact, it is common practice for a combination of the two to be used. Leach XS characterises and evaluates the environmental impacts of samples, in particular assessing releases of hazardous substances—mainly on the basis of leaching tests [16,17]. However, the Leach XS database in conjunction with the Orchestra system provides a tool by which to interpret the process by which hazardous substances are released. It allows obtained research results to be modelled, while also predicting leaching—temporally, and in relation to research space [16].

Fuzzy systems have proved their excellent ability to serve as system identification tools [18,19,20]. Their main advantage is the way they may work with a much more limited amount of information concerning the system [21,22,23]. The use of fuzzy rule-based systems (FRBS) in system identification can be seen as an approach by which systems are modelled using fuzzy-logic descriptive language with fuzzy paradigms [24]. The paradigm in question has a proven ability to generate various kinds of fuzzy models automatically from data, allowing human expertise to be incorporated, with integration of numerical and symbolic processing into a common schema [25].

Performed laboratory tests often supply readings with a small number of results, so that ensures the importance of using significant non-quantitative knowledge about a system, in order for identifiable models to be obtained. The fuzzy system has been used in geological research already [26] as well as in regard to the leachability of elements from building materials [27]. However, these studies were not conducted in the context of the leachability of strontium and barium. Now the work detailed here refers to the use of the Mamdani–Assilian model [28] in preparing a system concept.

### 1.1. The Geochemical and Toxicological Characteristics of Strontium (Sr) and Barium(Ba)

Strontium and barium are among trace elements listed as potential standard chemical markers for building materials. European data from CEMBUREAU [29] augments information on strontium and barium with more concerning Mn, Mg, Zr, Ti, V and Zn [30,31]. Sr and Ba are elements in group II of the periodic system, and manifest oxyphilic properties. There is a geochemical similarity between strontium and barium due to the similar size of the ion rays.

Strontium is a common element, present in the Earth’s crust at concentrations ranging from 150–480 mg·kg^−1^ (mean 350) (Table 1). The element most often goes into solution in the form of a bicarbonate generated during weathering processes. It is easily absorbed by clayey minerals, as well as organisms that form carbonate skeletons. Its concentration is greater in sedimentary rocks, in particular carbonates and clay rocks. The average concentration in the former is 610 mg·kg^−1^ (Table 1). The strontium content in soils is in the range of X0–X000 ppm, being closely related to the nature of the given bedrock. The strontium content in acidic soils is usually lower than where calcium carbonate is present. It also mostly increases with depth. The element is absorbed by plants in soils and limestone rocks [32].

The biogeochemical properties of strontium closely resemble that of calcium. The quantitative ratio of these elements are used as an index unit to determine the dispersion or concentration of strontium in certain ecosystems. Strontium 90 (^90^Sr)—radionuclide, is hazardous to the biological environment and is also of great geochemical importance. The accumulation of radioactive strontium in soils poses the threat of organisms becoming contaminated as incorporation into the food chain takes place. The isotope is readily digestible by plants, animals and humans, going on to concentrate in the bones and milk of mammals. In light soils strontium 90 (^90^Sr) becomes partially dissolved in water, and thus poses a major threat to the natural environment and human health. 

Weathering of minerals containing strontium ensures the presence of the element in solution, e.g., in bicarbonate, chloride, sulphate or complex-compound form. 

In the organisms of animals and human beings, strontium is involved in the metabolism of calcium and phosphorus. In excess, it leads to decalcification and bone deformation [33].

Barium in the Earth’s crust is on average present at 628 mg·kg^−1^. The element proves to be more concentrated in the continental part of the Earth’s crust than strontium and occurs markedly more frequently (Table 1). In terms of geochemical relationships, barium differs from strontium in that its ionic radius (of 1.43 Å) is such as to allow it to bind diadochically with potassium (even as there is no such relationship with calcium). As a result of this diadochia, barium is mainly present in rock-forming potassium minerals, especially in feldspars. However, a second rock-forming mineral containing considerable amounts of barium is biotite. As a result of weathering, barium becomes dissolved in the form of bicarbonate. In a hypergenic environment, it is activated easily, then precipitates out as sulphates and carbonates. The commonest compound arising out of the precipitation of barium is BaSO_4_- barite. 

Comparison of the geochemical behaviours of barium and strontium in surface environments reveals much more limited mobility of the former, which precipitates out much more readily from surface-water environments. Consequently, barium becomes very much more diluted in seawater than does strontium. The very low concentration of barium in seawater eliminates the possibility of its becoming concentrated in salt deposits [34].

Moreover, barium becomes strongly sorbed by clayey deposits, manganese concretions and various compounds of sulphur. It concentrates mainly in clayey rocks, especially in deep sea sediments. Clastic rocks vary markedly in their contents of barium. An average figure for sandstones would be 20 mg·kg^−1^ (Table 1). Carbonate rocks are the group of sedimentary rocks poorest in barium (Table 1). Moreover, the Ba content in soils ranges from 175 to 520 mg·kg^−1^, while that in arable crops is in the range 1.5–170 mg·kg^−1^. Some marine organisms are found to have up to 700 mg·kg^−1^ Ba, while human blood has up to 2.4 µg L^−1^. Barium is toxic to plants at a concentration of 1–2% (by dry weight). For human beings, the LD50 lethal dose of barium chloride is of around 14 mg·kg^−1^. Acute toxicity is manifested in disorders of the cardiovascular, nervous and digestive systems. Death from poisoning by this element is caused by failure of the heart or respiratory system. It should also be emphasised that all soluble barium compounds are poisonous [33].

### 1.2. Geological Setting

**Figure 1:**
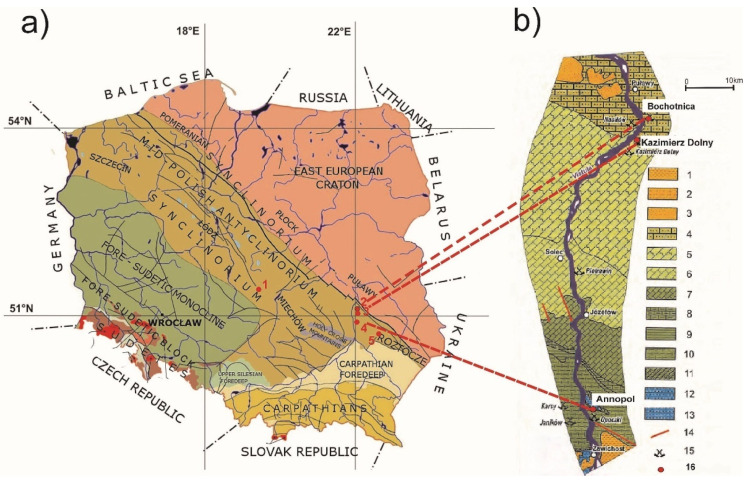
(**a**) Locations of sampling sites against the background of the main tectonic units in area of Poland [47]. Explanations: 1—Bełchatów; 2—Kazimierz Dolny; 3—Bochotnica; 4—Annopol; 5—Krasnobród; (**b**) Geological outline of the Middle Vistula Valley [42,48]. Explanations: 1—Miocene Krakowiec clays, 2—Miocene Lithotamnia limestones and sands, 3—Oligocene quartz and glauconitic sands, 4—Danian Green sand, 5—Upper Maastrichtian sandy or glauconitic marls and opoka rocks, 6—Lower Maastrichtian marls and opoka rock, 7—Campanian marls and opoka rock, 8—Santonian marls and opoka rock, 9—Coniacian and Santonian—glauconitic marls and opoka rock, 10—Turonian sandy limestones and opoka rock, 11—Albian and Cenomanian—quartz and glauconitic sands, 12—Kimmeridgian marls and oolite limestones, 13—Lower Kimmeridgian (Astart)-oolite, coral, quartz and glauconitic plate limestones, 14—fault, 15—abandoned quarry, 16- sampling points.

These are present commonly in the Mesozoic marine series, especially the Upper Cretaceous. They form interbeds with marls and limestones. Opoka rocks in Europeare present in all Upper Cretaceous levels. In Poland, their occurrence is common in vast areas of the Lublin Upland, in the Mogilno-Łódź and Nidziański Basins (Figure 1). In Poland, opoka rocks occur in the Mesozoic deposits: Maastrichtian, Santonian, Campanian, Cognac and Turonian. They form thick series with a thickness exceeding 600 m [39]. 

The Turonian opoka rocks from the vicinity of Annopol (Figure 1b) form layers with a thickness of about 200 m. The lowest part of the Lower Turonian in this area is formed in the form of gaizes and opoka rocks as well as hard, sometimes lumpy organodetritic limestones. Fine to medium shoal opoka rocks with cherts and levels of black flints were included in the Upper Turonian. The Turonian opoka rocks contain numerous fossils, the most abundant of which are inocerams, including *Mytiloides hercynicus* (Petrascheck) and *Inoceramus lamarian parkinson* —the predominant species for the Middle Turonian [40]. In addition, brachiopods, echinoderms and ammonites have been found. Foraminifera and calcareous nannoplankton are also numerous in the opoka rocks. The Upper Turonian is represented by white, plate-separated opoka rocks with a high content of glauconite and numerous inoceram shells [40]. 

The opoka rocks collected from the sites in Kazimierz Dolny, Bochotnica, Karsnobród and Bełchatów represent the Maastrichtian stratigraphy. In the area of Kazimierz Dolny, the opoka rocks form thick-layer complexes, which constitute over 60% of sediments in the profile of this geological floor. The remaining material in the exposed areas consists of limestones, marls and cherts (Figure 1b). Above them, locally the oldest Neogene formations have survived, formed as gaizes with limestone and marl inserts, with a thickness not exceeding 20 m. Well-preserved exposures of these formations known from the vicinity of Kazimierz Dolny are unique on a European scale. Quaternary formations, covering a large part of the area in question, lie directly on the Upper Cretaceous sediments or locally on Neogene formations. The oldest among them are sands with gravels and river silts of the Pre-Pleistocene and boulder clays of the South Polish glaciation, found only in boreholes. The sediments of the Central Polish glaciation are represented by clays, silts, bog and glacial sands, gravels, glacial boulders and boulder clays [41,42].

Apart from Poland, we find opoka rocks in the Czech Republic, Slovakia, Lithuania, Ukraine, France, The Netherlands and England. In Germany, opoka rocks can be found in Saxony in the Dresden region and in Westphalia. Their age is estimated at the Cenomanian and Turonian. In Lithuania, this stone was mined in the southern mining district of Stoniškiai, while in the Czech Republic rich deposits of opoka rocks extend from Kadań, east of Prague across to the Moravia region [43,44]. These rocks are also common in Russia among the lower Neogene sediments in the Volga Basin, on the eastern slopes of the Urals, in the Upper Cretaceous deposits of the Eastern European part of Russia, to the Cretaceous sediments of the Paris basin [45,46]. 

### 1.3. Materials

As part of the fieldwork, material for research was collected from five areas of Poland (Figure 1). Samples were taken from openings and quarries including the Bełchatów lignite deposit, at which opoka rocks from the Mesozoic/Neogene contact zone were exposed in the course of open-cast mining. A total of 110 rock samples were tested. 

**Figure 2:**
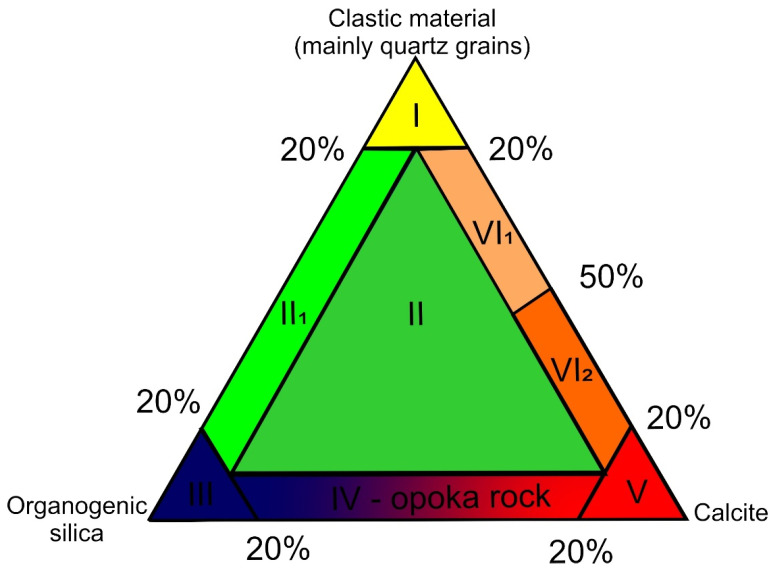
Position of the opoka rocks in the classification triangle: siliceous rocks—clastic rocks—calcareous rocks ([56], slightly changed). I—sands, sandstones, II—gaizes, II_1_—calcium—free gaizes, III—siliceous rocks, IV—opoka rocks, V—limestones, VI_1_—sandy and calcareous rocks, VI_2_—calcareous and sandy rocks.

The term opoka rock was first used in Poland in the 18th century. It referred to the mining description of Upper Cretaceous marls [49]. These rocks are described as Upper Cretaceous sediments [50,51,52,53,54]. Opoka rocks were defined as calcareous-silica rocks with an opal skeleton [55]. In petrographic literature, the term opoka rock is used to refer to a group of transitional rocks. In the group of these rocks, opoka rocks are defined as intermediate rocks between carbonate and siliceous rocks, apart from them there are marls—intermediate rocks between carbonate and clay ones. Types of transition rocks between siliceous, crumb and carbonate rocks—similar to opoka rock are gaizes [56]. Opoka rocks are distinguished by cryptocrystalline silica dispersed among carbonate components. Their characteristic feature is the presence of opal and chalcedony, forming highly developed skeletal structures. The opoka rocks are generally slightly lighter than the marl, often with a bluish tinge. There are sometimes pyrite concretions, marcasite concretions and flint nodules. They are widespread in marine sediments, especially Mesozoic ones [56]. The term transitional rock in reference to opoka rocks is given in other works. Kozłowski [57] describes transitional rocks between carbonate and siliceous rocks as opoka rocks. They are characterized by a significant amount of dispersed cryptocrystalline silica. The opoka rocks contain 5–75% of autogenous silica in the form of opal or chalcedony. These rocks, digested in hydrochloric acid, do not disintegrate due to the presence of a silica skeleton [57]. In the work of Manecki et al. 2008, in the subsection “transition rocks from siliceous rocks to other rock groups and with a mixed mineral composition”, opoka rocks are a transitional formation between siliceous rocks and limestones. They are made of calcite and a subtle skeleton of autogenous silica (opal, less often chalcedony), often also organic remains (especially sponge needles) [58]. The position of the opoka rocks in the international petrographic classification of sedimentary rocks (Figure 2).

**Figure 3:**
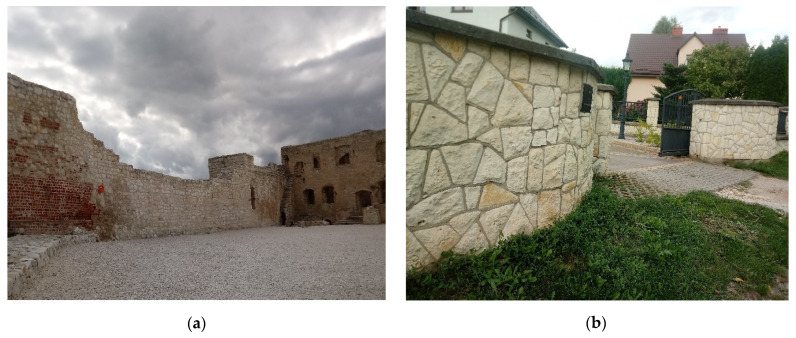
(**a**) The walls of the Castle in Kazimierz Dolny on the River Vistula, made of opoka rock. 14th century; (**b**) Contemporary use made of opoka rocks from the Bochotnica region (author’s own elaboration).

Opoka rocks are used in construction and as road stone. Castles, churches and houses have been erected using this white stone. Currently, this a material that also gains common use as a decorative facade and cladding material (Figure 3a,b). It is used in the production of Portland clinker, to make cement. The porosity of the opoka rock is high (at around 42% on average), and this has a positive effect on the thermal resistance displayed by walls made of this stone. The thermal conductivity of these rocks is of approximately λ = 0.61 W/m^2^·K. This value brings the material closer to the properties of building ceramics and lightweight concretes. This material is characterised by a relatively low specific gravity, with the average value for the studied deposits being about 1.46 g/cm^3^ in Poland’s Kazimierz Dolny region, and over 2.0 g/cm^3^ in the cases of Bochotnica and Krasnobród. This has meant that even quite large blocks can be moved and processed using human physical strength. An additional characteristic feature of the opoka rocks is that they are easy to work when freshly excavated. Their features, such as bright colour and ease of cleaning (as even rainwater washes dirt off it) ensure that buildings made of them are plastered only rarely. The popularity of opoka rock as a masonry material was mainly influenced by its availability. In many places, deposits lie superficially under the humus layer.

In turn, negative features of opoka rocks include their low compressive strength (of average value 38.18 MPa). In addition, the strength of these rocks drops sharply to 50% when in contact with water. Compared to carbonate rocks, they are characterised by low abrasion resistance. They also display high levels of water absorption of up to 50%, as well as only limited frost resistance [59,60].

## 2. Methodology

Water was extracted from samples of monolithic opoka rock, in accordance with the NEN 7375 standard from 2004 [61]. Strontium and barium leaching was undertaken in relation to 8 time fractions: 0.25 days (6 h); 1 day (24 h); 2.25 days (54 h); 4 days (96 h); 9 days (216 h); 16 days (384 h); 36 days (864 h) and 64 days (1536 h). Relevant tests were carried out at temperatures of 18–22 °C. The mineralisation of water eluates was achieved in line with the PN-EN ISO 11885 standard; while concentrations of strontium and barium were as determined using the ICP–OES sequential plasma emission spectrometer. The concentrations of strontium and barium were determined using a sequential ULTIMA 2 HORIBA JOBIN-YVON plasma ICP–OES spectrometer (Longjumeau, France, with the possibility of retrospective analysis, and operating in the spectral range 160 to 800 nm, with the possibility of extension to 120–800 nm at any time. The software cooperating with the ICP–OES spectrometer allows for registration of the full spectrum in less than 200 s, with full resolution of the spectrometer. The study of quantitative concentrations of strontium in water eluates was carried out at the Aerospace Materials Research Laboratory of Rzeszów University and Technology. The laboratory is accredited by Nadcap (the National Aerospace and Defense Contractors Accreditation Program), and issued in March 2009 by the Performance Review Institute, and the US and Polish Centres for Accreditation [62].Observations of the opoka rocks’ microstructure were made using scanning electron microscopy (SEM) methods, as combined with the use of an X-ray energy dispersion microscope (EDS). This made it possible to analyse the chemical composition of the samples in micro-areas. For the purposes of SEM, use was made of an FEI Quanta 200FEG electron microscope (Hillsboro, OR, USA) equipped with an X-ray spectrometer (EDX Genesis, and a backscattered electron detector (BSE).

Mineral phases were identified using a PHILIPS X’PERT PW 3020 X-ray diffractometer (Malvern, UK) using Ni-filtered Cu Kα radiation. Diffraction images were recorded across a 30°–70° range of angles (Θ).

Microscopic observations in reflected light along were carried out using a Motic Panthera TEC POL trinpolarising microscope (Hong Kong, China). The micrographs were taken using a high-sensitivity microscope camera with as CMOS matrix and a Motic Globar Shutter type Pro-S5 Lite shutter (Hong Kong, China).

Cathodoluminescence analyses (CL) were performed using a Cambridge Image Technology Ltd. Model CCL 8200 mk^3^ (Cambridge, UK). This was coupled with an Optiphot 2 microscope equipped with a Canon EOS 600D digital camera (Tokio, Japan). Tests were carried out on exposed hard plates with a polished surface. Heat-resistant resin was used to make these.

### Calculation Method/Numerical Analysis for the Sr and Ba Leaching Model

**Figure 4:**
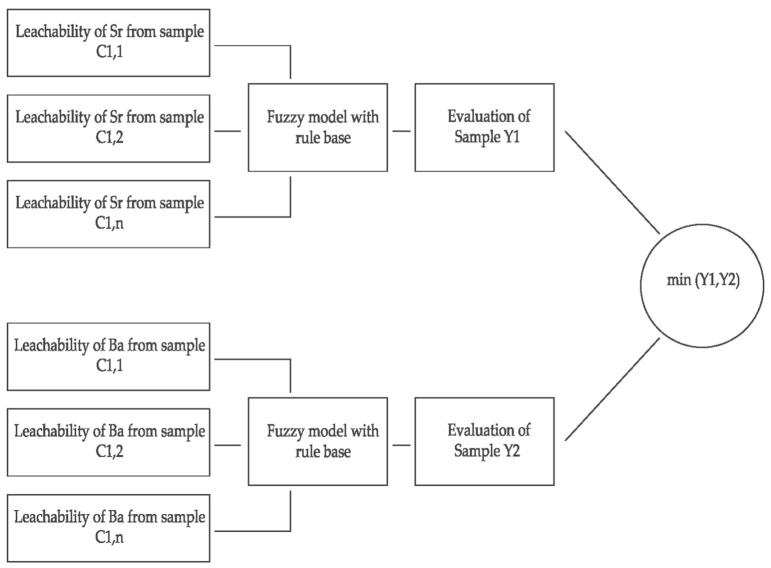
General scheme of fuzzy inference of the elution of Sr and Ba from opoka rock.

The assessment of the leachability of heavy metal elements Sr, Ba was performed using the fuzzy model due to the fact that there are no sharp boundaries between the values of the variables under consideration. The advantage of using fuzzy models, compared to conventional mathematical models, is that there is no need to have a large database of results about the system, e.g., empirical results. Moreover, the system information may be imprecise in nature. An additional argument for the use of fuzzy modeling is the possibility of its use when the relationships between the input and output quantities are difficult to describe with mathematical, statistical [63] or numerical [64] relationships. Fuzzy inference is realized through the use of logic, actions on fuzzy sets and expert knowledge concerning the studied phenomenon, e.g., leachability of heavy metals from the rock. In the conducted analysis, three linguistic variables were introduced: small, average, good. In turn, the values of linguistic variables are assigned fuzzy sets, while the relationships between linguistic variables are fuzzy conditional tasks. Figure 4 shows the general scheme of inference fuzzy leachability of Sr and Ba from the rock.

Determination of the relationship between the input quantities of the leaching of Sr and Ba from the opoka rock as well as the value for quantitative output over successive time steps was obtained thanks to the use of a decision block. In the one under consideration, fuzzy models were used, given the nature of the analysed data. It is related to the necessity to evaluate rock samples for the leachability of Sr and Ba over time, as related to the lack of sharp boundaries between values for the variables. The choice of fuzzy models reflects the more-limited information regarding the system, as compared with conventional mathematical models. Fuzzy inference is used in the construction of numerical models when the relationship between the input and output quantities cannot be described by mathematical equations or is difficult to determine using standard statistical [64] and numerical software [65]. To perform the calculations, the Mamdani–Assilian model was used as part of the Fuzzy Logic Toolbox add-on, operating in the MATLAB environment (R2018b) [66]. The fuzzy inference in this case under consideration is based on the assigning of “linguistic” quantities to values for the input and output variables. In turn, specific fuzzy sets were assigned to linguistic values. The whole procedure of fuzzy inference in the discussed MATLAB environment in fact consists of 5 stages:

- Definition of the membership function;
- Fuzzification;
- Knowledge representation;
- Inference;
- Defuzzification [65].

## 3. Results

### 3.1. Empirical Resultes

**Figure 5:**
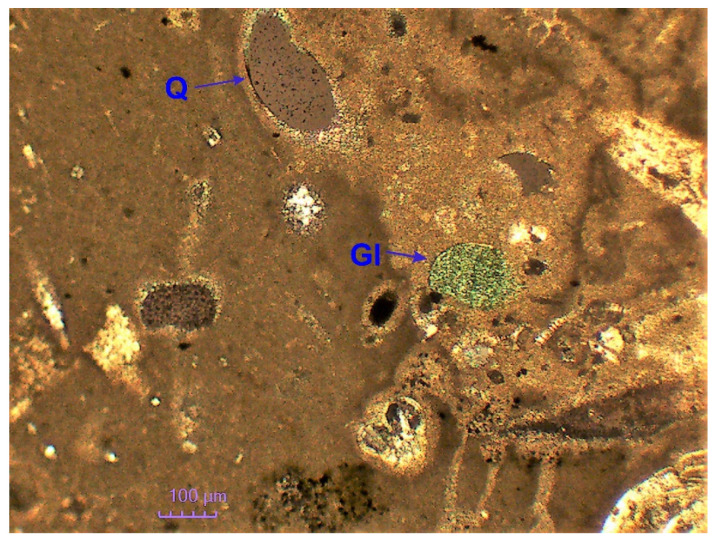
Micrite-organic structure of opoka rocks. Polarizing microscope, Xp. Xp—crossed polarizers, Q -quartz, Gl—glauconite.

**Figure 6:**
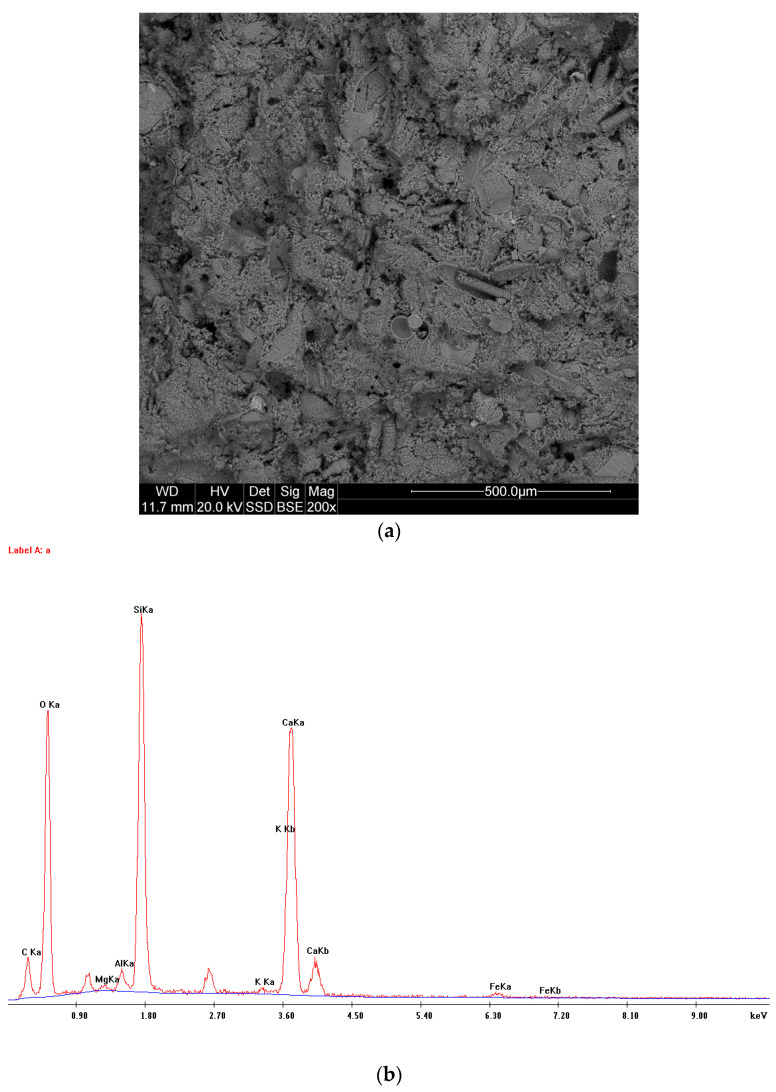
(**a**) The silica-carbonate structure of the opoka rocks under electron-microscope imaging (SEM). SEM—scanning electron microscopy. (**b**) Analyses of the chemical composition of the samples (EDX). EDX—electron microscope equipped with an X-ray spectrometer.

**Figure 7:**
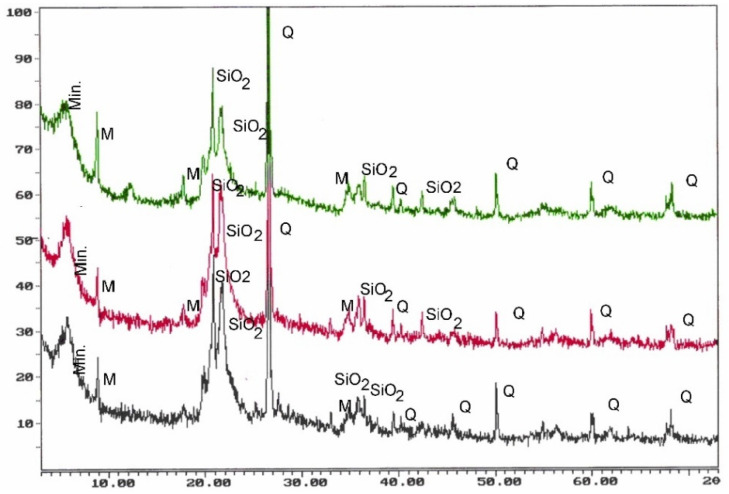
Examples of diffractometric curves. SiO_2_—silica phases in the form of opal (CT varieties relative to C), Q—quartz, M—mica, Min.—clayey minerals [67].

**Figure 8:**
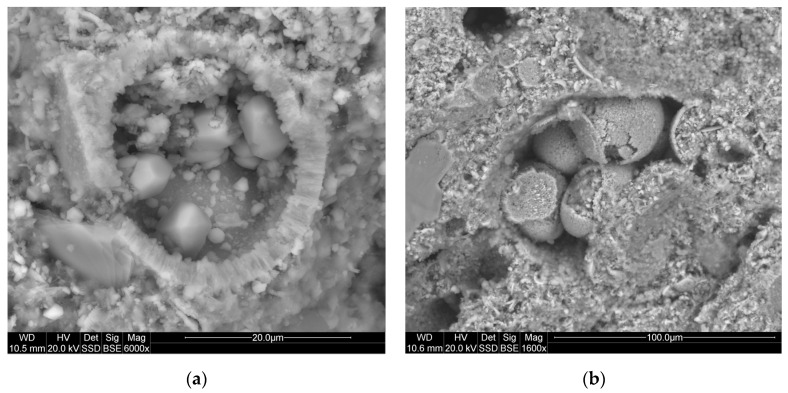
(**a**) Well-preserved quartz crystals in oval bioclast void; (**b**) Framboid pyrite in the voids of bioclasts. Image SEM. SEM—scanning electron microscopy.

**Figure 9:**
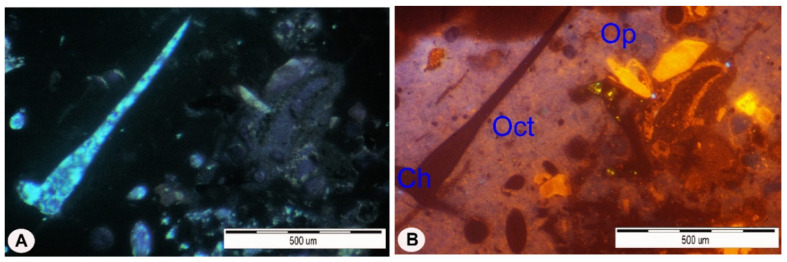
The opoka-rock: (**A**) Polarizing microscope, Xp; (**B**) CL image revealing an opal rocky background–pink luminescence, and relics of feldspar and carbonate. Xp—crossed polarizers, CL—cathodoluminescence, Ch—microcrystalline chalcedony, Oct—opal cristobalite–tridymite, Op—Opal [67].

Mineralogical studies have shown that the studied rocks most often have a micrite-organogenic structure, and less frequently a micrite—detritic structure (Figure 5). Texture is slightly porous (Figure 6a). The main mineral component of opoka rock is silica (Figure 6b). It has been possible to distinguish opoka rocks in which silica is in the form of opal type A and type CT (cristobalite—tridymite) (Figure 7). In addition, in amounts of up to 10% of the opoka rocks, detrimental quartz grains of silt and clay fractions could be found. Well-formed quartz crystals were observed in oval bioclast voids (Figure 8a). Cathodoluminescent analyses confirm the presence of different generations of silica. The oldest take the form of brown-luminescence quartz. The CL image mainly shows chalcedony-building no luminescent sponge needles. Blue-luminescence opal-CT is observed in bioclasts. Silica of pink-blue luminescence constituting the rock matrix suggests its opaline nature. It is the youngest generation of silica (Figure 9B). Penetrating primary calcite cement of red luminescence is visible in the observed rock matrix [67].

Apart from silica, carbonate minerals proved to be a key component. Calcite in the form of micrite forms the rocks background, while carbonates built up numerous organic debris, mainly represented by the shells of foraminifera. Opal bioclasts represented by sponge needles have also been found. The accessory minerals of the opoka rocks were formed by single muscovite plates, glauconite, dispersed plant carbonaceous substances (Figure 5). Pyrite was dispersed and aggregated in some bioclasts (Figure 8b). Heavy minerals were represented by zircon, rutile and tourmaline. Among the accessory minerals only those of the clayey and opaque ore kinds could be found in the decalcified opoka rocks.

**Figure 10:**
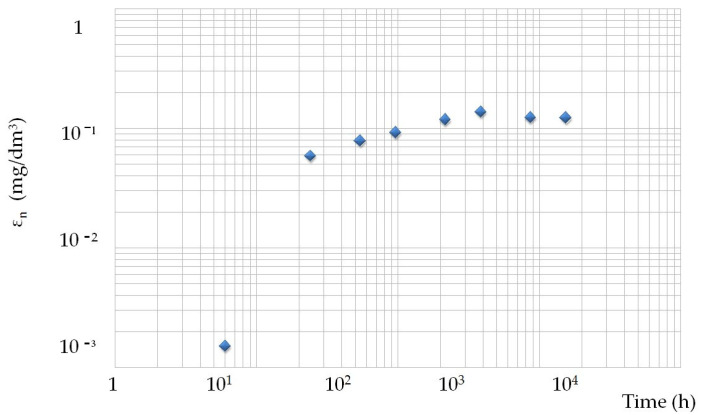
Total leachability of strontium from the opoka rock over time. ◊—average leachability of Sr from opoka rock (mg/dm^3^).

**Figure 11:**
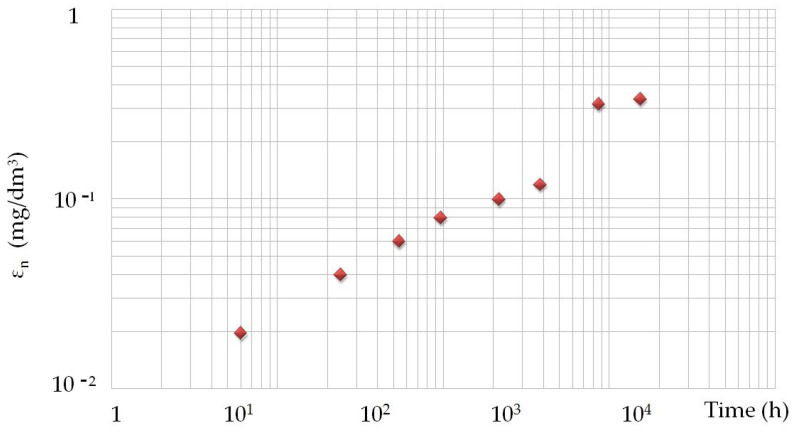
Total leachability of barium from the opoka rock over time. ◊—average leachability of Ba from opoka rock (mg/dm^3^).

Opoka rock leachability showed an upward trend in the first months. Concentration in eluates increased steadily, with the highest value of 0.14 mg/L achieved at 384 h. After 16 days, the concentration decreased gradually to reach a value of 0.12 mg/L. The leachability of barium did not show any significant changes over time; the result remaining at almost the same level of 0.019 mg/L (Table 2). The presented empirical values of leachability Sr and Ba are simultaneously the input quantities of the fuzzy inference analysis (Table 2). The results of the total leachability over time are presented in Table 3. In the graph (Figure 10) it can be seen that the strontium leachability was the fastest after 24 h. In subsequent time intervals strontium was leached out evenly. After 1536 h, the leachability of strontium drops significantly. The graph of the total barium leachability against time shows the linear nature of this process. After the time, the decrease in the bar’s leachability in 1536 h is slight (Figure 11).

### 3.2. The Model forSr and Ba Leachability

Due to the lack of sharp boundaries between values for individual variables, the fuzzy model was used to evaluate samples in terms of leaching of the elements. This was achieved in line with a procedure involving the following steps.

#### 3.2.1. The Definition of Membership Functions Used in the Model

The model is based on two types of membership functions:

- The Gauss curve, Equation (1):
  (1) $$\mathrm{gauss}\left(t,\;\sigma ,\;m\right)=e^{\frac{-{\left(t-m\right)}^{2}}{2\sigma^{2}}}$$
  where:
  σ—standard deviation,
  m—the expected value,
  t—the independent variable.
- The sigmoid curve, Equation (2):
  (2) $$\mathrm{sig}\left(t,\;a,\;c\right)=\frac{1}{1+e^{-a\left(t-c\right)}}$$
  where:
  a—growth rate,
  c—the inflection point,
  t—the independent variable.

#### 3.2.2. Fuzzification

As part of the fuzzification stage, conversion of input variables to the fuzzy domain, Gauss and sigmoid curves presented by Equations (1) and (2) were used.

- Two input variables for the leachability of Sr and Ba (predecessor linguistic variables);
- One output variable Y (the successor linguistic variables input variables).

The functions proposed above are presented in Table 4 below, along with ranges for the variability of input quantities (real and linguistic).

**Figure 12:**
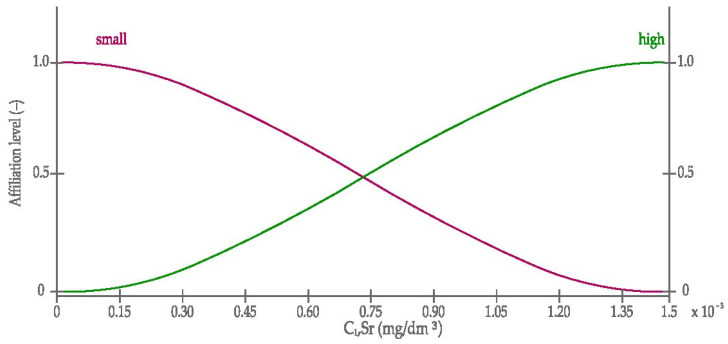
The sigmoid curves used in modelling Sr leaching from opoka rock. C_1,_Sr—leachability of Sr.

**Figure 13:**
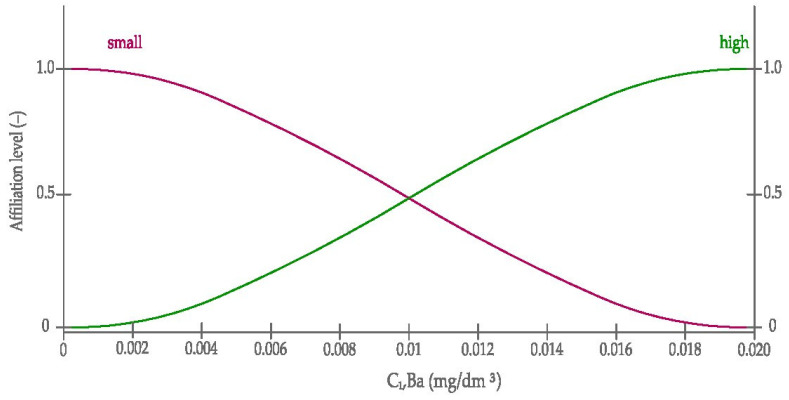
The sigmoid curves used in modelling Ba leaching from opoka rock. C_1,_Ba—leachability of Ba.

**Figure 14:**
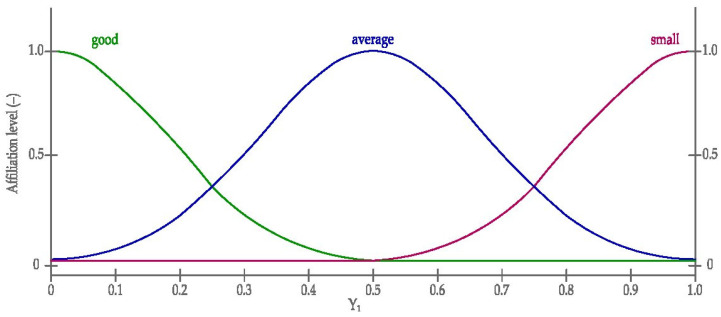
The sigmoid and Gauss curves used in modelling output quantity. Y_1_—diagnostic assessment of the value of the output variable.

Graphic diagrams of sigmoid curves, according to the Equation (2) of the linguistic (input) variables belonging to the fuzzy inference model are presented in Figure 12 and Figure 13. On the other hand, the sigmoid and Gaussian scheme, according to the Equations (2) and (1), of belonging to the linguistic (output) variable of the fuzzy inference model is presented in Figure 14.

#### 3.2.3. Knowledge Representation

For each of the eight analyzed samples, four rules of the expert system were defined. Table 5 below shows the rock samples used in fuzzy reasoning considered in one of the time steps.

#### 3.2.4. Inference

**Figure 15:**
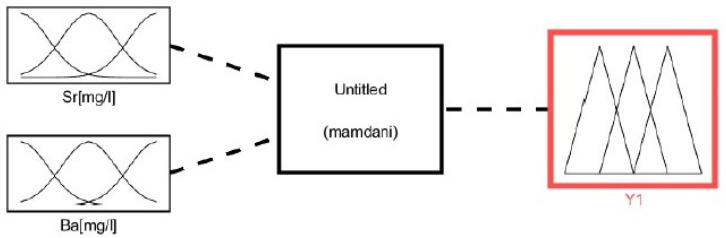
Diagram showing the numerical model applied.

The Mamdani–Assilian model in the MATLAB environment was used to solve the problem of the inference of the fuzzy leachability of Sr and Ba from the rock. In the part of the analysis related to inference, the rules for inference of the input linguistic variables presented in Table 5 were applied. The fuzzy inference process conducted in the MATLAB environment was carried out in accordance with the scheme presented in Figure 15 and with the use of linguistic operators presented in Table 6.

#### 3.2.5. Defuzzification

The process of defuzzification in the MATLAB environment, i.e., the process of refining, converting fuzzy sets to the crisp domain, was carried out according to the mean values of the centers of gravity of the response surface according to the equation:

(3) $$Y=\frac{\sum_{l=1}^{M}c_{l}\cdot \mu_{F^{\left(l\right)}\left(x^{\left(l\right)}\right)}}{\sum_{l=1}^{M}\mu_{F^{\left(l\right)}\left(x^{\left(l\right)}\right)}}$$

where:

c_l_—fuzzy set center,
μ_F(l)_—function of membership of fuzzy sets F^(l)^ corresponding to a given input variable.

**Figure 16:**
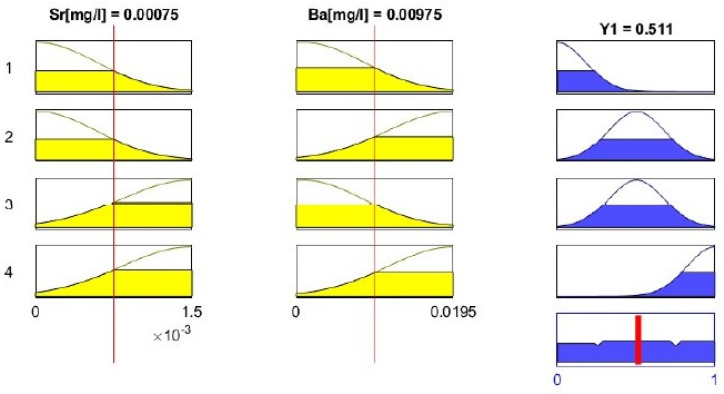
The inference procedure conducted within the MATLAB environment.

An exemplary fuzzy inference image carried out in a MATLAB environment, for the leaching period of 6 h, which corresponds to the empirical results of leaching [mg/dm^3^] Sr (0.0015) and Ba (0.0195), is presented in Figure 16.

The results of the performed defuzzification for all analyzed time steps of elution of elements and the input variables adopted in the analysis are presented in Table 7.

**Figure 17:**
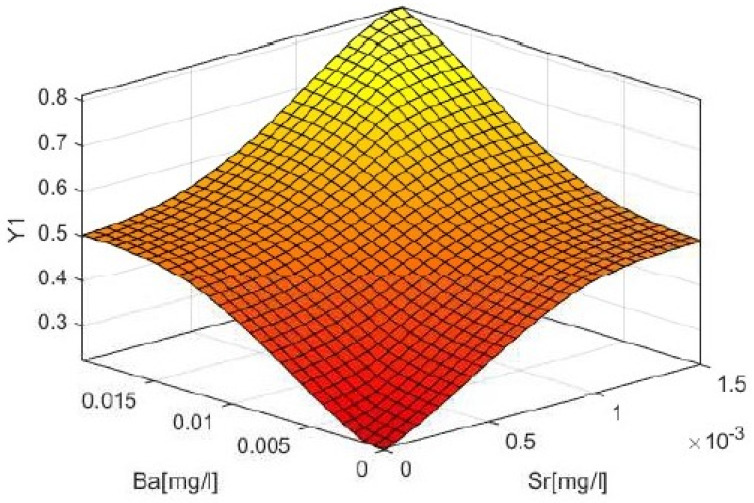
The plane–form relationship for the response of the output variable Y1 to the input variables (leachability of Sr and Ba after 6 h).

Simulated values of the linguistic variable of the output variable in the analysis—as based on values for the input linguistic variables—were obtained through use of the centroid function, which allows the center of gravity of a surface to be determined. Figure 17 offers a graphic presentation of the response surface for satisfaction level Y, in relation to values for the leaching of Sr and Ba over the considered time step.

Table 8 presents results obtained for fuzzy inference, the result function Y, and the leaching of Ba and Sr from opoka rock, using the Mamdani–Assilian model.

The results obtained as part of the fuzzy processes of fuzzy inference prove the “average” assessment of the inference system. For this reason, while maintaining the expert opinions of the conducted analyzes, the Mamdani–Assilian model can be used to assess the leachability of Ba and Sr in the considered range of input variables. The undoubted advantages of the model used are: the stability of the model, ease of expression and interpretation of the obtained results and the possibility of limited interpolation of the output variable for input variables outside their initial range.

## 4. Discussion

The natural cycle of trace elements in nature is usually characterized by an equable balance between the amount of elements released as a result of hypergenic processes and their binding in geological formations. All chemical elements activated as a result of various human activities are subject to only a certain extent to various forms of migration and gradually, partially included in the natural cycle. The development of industry, urbanization and transport influences directly or indirectly the chemical changes of food products, air and water, which are decisive for human health. The harmfulness of the trace elements polluting the environment is largely due to their biochemical and biological properties. In the case of the analysed elements, strontium is susceptible to bioaccumulation from the aquatic environment, and barium concentrates in biolites as a result of geological processes. With a weakened effect of biological barriers, these elements accumulate cumulatively, which results in their constant accumulation in the last link of the food chain—human. Strontium accumulates in tissues and organs such as bones, aorta, testes, prostate, while barium accumulates in the skin, lungs, bones and teeth. The main elements of the environment from which trace elements pass into living matter are soil, water and air. Among the various elements of the biosphere, soils occupy a special position because not only they are the main center of accumulation of many chemical pollutants, but they also act as a protective filter for both components migrating to the water and for easily volatile elements. The greatest changes in the chemical composition of soils occur most often in the immediate vicinity of industrial emitters. Among the industrial plants which are the main sources of contamination with strontium and barium, apart from the chemical and pulp and paper industries, the ceramic, cement and glass industries are mentioned. In the current research work in the eastern EU buffer strip, areas with a high concentration of trace elements, mainly strontium, in soils adjacent to cement building materials production plants were observed [68]. The studies of Sr content in the opoka rocks in central Poland showed concentrations at the level of 396–223 mg·kg^−1^, with an average value of 293 mg·kg^−1^ [69]. The content of the investigated trace elements in the soils in the study area was at the level of 3–560 mg·kg^−1^ for strontium, with an average value of 13.32 mg·kg^−1^ and for barium at the level of 7.3–225.7 mg·kg^−1^ with an average value of 49.56 mg·kg^−1^. The determined total leachability of the examined elements in the time of 1536 h was 0.7320 mg/L for strontium and 0.3351 mg/L for barium (Table 3). Comparing the obtained results with the content of strontium and barium in the waters of the World and Europe (Table 1), it can be noticed that the values of both determined elements are exceeded. On this basis, it can be assumed that in the vicinity of the opoka rocks, the effects of water will result in a cumulative concentration of strontium and barium. However, it should be emphasized that the obtained results of strontium and barium concentration do not exceed 0.1 mg/L, the permissible content in drinking water.

The use of fuzzy inference in this study was to verify and justify the use of this procedure to predict the leachability of Ba and Sr from the rock. One of the important premises prompting the authors to choose the fuzzy inference procedure was the possibility of using it with a small amount of measurement data. In addition, the stability of the obtained solution and the possibility of determining the parametric level of the leachability of Sr to Ba at individual measuring moments increases the range of its applicability.

The results obtained for fuzzy inference, result function Y, and the leaching of Ba and Sr from opoka rock, in line with empirical data obtained, point to “average” assessment of the inference system for the experiments conducted. An “average’’ result for the variable Y result was obtained for each time step considered in the work. There were no anomalies or step changes characterizing the value of the resultant leaching function for the experiments performed. For this reason, the obtainment of an “average” result permits use of the Mamdani–Assilian model in assessing the leachability of Ba and Sr within the range of values. Additionally, and with a view to the credibility of the expert result obtained from the model being increased further, the scope of the experiment base should be extended and compiled together with other independent data analyses. The unquestionable advantages of the fuzzy inference method used in this work reflect the possibility of its being applied with many variants of experiments, the short time needed for results to be obtained, and the possibility of extension by reference to further input variables, providing toward the prediction of potential stabilization effects related to the leaching of elements from opoka rock as a function of time.

The obtained and discussed results of fuzzy inference justify the use of this computational procedure with an acceptable level of fit. This enabled the determination of the response surface showing the changes in the leachability of Sr and Ba relative to each other from the rock at particular time steps. For this reason, the conducted analysis and the obtained results constitute an additional utilitarian goal of the work as a useful, verified tool that can be used in subsequent scientific works.

The development of the proposed expert system requires a lot of experimental work and will be the subject of further scientific work by the authors.

## Figures and Tables

**Table 1:** Literature-based summary on the mean values of strontium and barium in the environment.

Origin-Source		Median (mg·kg^−1^)		
		No. of Samples	Strontium (Sr)	Barium (Ba)
Crust [35]	upper continental	n.a.	350	628
Soil [36]	World	n.a.	240	500
Subsoil [37]	Europe	788	95.0	385
Topsoil [37]	Europe	845	89.0	375
Water [37]	World	n.a.	0.50 (mg L^−1^)	30 (μg L^−1^)
Water [37]	Europe	807	0.11 (mg L^−1^)	24.9 (μg L^−1^)
Carbonate rocks [38]	World	n.a.	610	10
Sandstones [38]	World	n.a.	20	10
Humus [37]	Europe	367	17.4	60.6

**Table 2:** The mean rates of leaching of Sr and Ba from opoka rocks over successive time steps.

Time [h]	Average Leachability of Elements from Opoka Rock (mg/L)	
	Sr	Ba
6	0.0015	0.0195
24	0.0587	0.0202
54	0.0785	0.0197
96	0.092	0.0197
216	0.1182	0.0197
384	0.1375	0.0198
864	0.1231	0.0197
1536	0.1225	0.0195

**Table 3:** Total leachability of elements from opoka rock.

Time [h]	Total Leachability of Elements from Opoka Rock (mg/L)	
	Sr	Ba
6	0.0015	0.0195
24	0.0602	0.0397
54	0.1387	0.0594
96	0.2307	0.0791
216	0.3489	0.0988
384	0.4864	0.1186
864	0.6095	0.3156
1536	0.7320	0.3351

**Table 4:** Ranges for the variability of input quantities (real and linguistic).

C_1-1,Sr_ (mg/L)	C_1-1,Ba_ (mg/L)	Y
Small: sig {−0.00075; 0}	Small: sig {−0.00975; 0}	Good: sig {−10; 0.5}
High: sig {0; 0.00075}	High: sig {0.00075; 0}	Average: gaus {0.2; 0.5}
		Bad: sig {10; 0.5}

**Table 5:** Application of the fuzzy inference rule.

No	The Rules of Fuzzy Inference
1	If (Sr (mg/dm^3^) is small) and (Ba (mg/dm^3^) is small) then (Y is good)
2	If (Sr (mg/dm^3^) is small) and (Ba (mg/dm^3^) is high) then (Y is average)
3	If (Sr (mg/dm^3^) is high) and (Ba (mg/dm^3^) is small) then (Y is average)
4	If (Sr (mg/dm^3^) is high) and (Ba (mg/dm^3^) is high) then (Y is bad)

**Table 6:** Application of linguistic operators during the process of fuzzy inference.

Linguistic Operator	Operator Use Case
Conjunction	Min
Alternative	Max
Implication	Min
Aggregation	Max

**Table 7:** Summary of the obtained values of the initial quantities for the empirically tested leachability of Sr and Ba from the rock.

Time	Cumulative Leachability of Metal (mg/dm^3^)		The Result of Defuzzification
(h)	Ci,Sr	Ci,Ba	Yi
6	0.0015	0.0195	0.511
24	0.0587	0.0202	0.500
54	0.0785	0.0197	0.500
96	0.092	0.0197	0.500
216	0.1182	0.0197	0.500
384	0.1375	0.0198	0.500
864	0.1231	0.0197	0.500
1536	0.1225	0.0195	0.500

**Table 8:** Summary of fuzzy-inference results in relation to the leaching of Ba and Sr from the opoka rock for all considered time steps.

Time (h)	Quantity for the Variable Y Result	
	Sr to Ba	Ba to Sr
6	average	average
24	average	average
54	average	average
96	average	average
216	average	average
384	average	average
864	average	average
1536	average	average

## Data Availability

Not applicable.
